# Transcriptome and proteome analysis reveals the anti-cancer properties of *Hypnea musciformis* marine macroalga extract in liver and intestinal cancer cells

**DOI:** 10.1186/s40246-023-00517-0

**Published:** 2023-07-31

**Authors:** Rodiola Begolli, Myrto Chatziangelou, Martina Samiotaki, Andreas Goutas, Sofia Barda, Nikolaos Goutzourelas, Dimitrios Phaedon Kevrekidis, Paraskevi Malea, Varvara Trachana, Ming Liu, Xiukun Lin, Nikolaos Kollatos, Dimitrios Stagos, Antonis Giakountis

**Affiliations:** 1 Department of Biochemistry and Biotechnology, School of Health Sciences, University of Thessaly, 41500 Biopolis, Larissa Greece; 2 B.S.R.C “Alexander Fleming”, 34 Fleming str, 16672 Vari, Greece; 3 Laboratory of Forensic Medicine and Toxicology, Department of Medicine, Aristotle University of Thessaloniki, 54124 Thessaloniki, Greece; 4 Department of Botany, School of Biology, Aristotle University of Thessaloniki, 54124 Thessaloniki, Greece; 5 Department of Biology, Faculty of Medicine, University of Thessaly, 41500 Biopolis, Larissa Greece; 6 Key Laboratory of Marine Drugs, Ministry of Education, School of Medicine and Pharmacy, Ocean University of China, Qingdao, 266003 China; 7 Laboratory for Marine Drugs and Bioproducts of Qingdao National Laboratory for Marine Science and Technology, Qingdao, 266237 China; 8 College of Marine Sciences, Beibu Gulf University, 12 Binhai Rd, Qinzhou, 535011 Guangxi China

**Keywords:** *Hypnea musciformis*, Marine macroalga (seaweed) extracts, Chemoprevention, Transcriptomics, Proteomics, Liver cancer, Intestinal cancer, p53

## Abstract

**Background:**

Marine seaweeds are considered as a rich source of health-promoting compounds by the food and pharmaceutical industry. *Hypnea musciformis* is a marine red macroalga (seaweed) that is widely distributed throughout the world, including the Mediterranean Sea. It is known to contain various bioactive compounds, including sulfated polysaccharides, flavonoids, and phlorotannins. Recent studies have investigated the potential anticancer effects of extracts from *H. musciformis* demonstrating their cytotoxic effects on various cancer cell lines. The anticancer effects of these extracts are thought to be due to the presence of bioactive compounds, particularly sulfated polysaccharides, which have been shown to have anticancer and immunomodulatory effects. However, further studies are needed to fully understand the molecular mechanisms that underlie their anticancer effects and to determine their potential as therapeutic agents for cancer treatment.

**Methods:**

*H. musciformis* was collected from the Aegean Sea (Greece) and used for extract preparation. Transcriptome and proteome analysis was performed in liver and colon cancer human cell lines following treatment with *H. musciformis* seaweed extracts to characterize its anticancer effect in detail at the molecular level and to link transcriptome and proteome responses to the observed phenotypes in cancer cells.

**Results:**

We have identified that treatment with the seaweed extract triggers a p53-mediated response at the transcriptional and protein level in liver cancer cells, in contrast to colon cancer cells in which the effects are more associated with metabolic changes. Furthermore, we show that in treated HepG2 liver cancer cells, p53 interacts with the chromatin of several target genes and facilitates their upregulation possibly through the recruitment of the p300 co-activator.

**Conclusions:**

Overall, the available evidence suggests that extracts from *H. musciformis* have the potential to serve as a source of anticancer agents in liver cancer cells mainly through activation of a p53-mediated anti-tumor response that is linked to inhibition of cellular proliferation and induction of cell death.

**Supplementary Information:**

The online version contains supplementary material available at 10.1186/s40246-023-00517-0.

## Background

Cancer is a leading cause of death worldwide [1]. According to the World Health Organization, on a global scale one in six deaths in 2020 was due to the development of neoplastic malignancies [1]. Thus, it is imperative to develop new and effective methods for cancer treatment. One of the most important strategies for fighting cancer is chemoprevention, defined as the use of natural or synthetic compounds as drugs or through the diet for the prevention or even the reversal of carcinogenesis [2, 3]. In recent years, intensive research interest for anticancer compounds found in marine organisms has been performed. Such compounds present high structural and functional diversity, acting a rich source of natural chemical compounds [4, 5]. Moreover, incidence of specific cancer types is low in populations with a high intake of seafood [4] Among marine organisms, seaweeds have been shown to contain various chemo-preventive or chemotherapeutic compounds, possessing different mechanisms for inhibiting carcinogenesis [6, 7].

*Hypnea musciformis* (Wulfen) J.V. Lamouroux (Gigardinales: Rhodophyta) is one of the seaweeds whose compounds have been reported to possess anticancer properties [8, 9]. *H. musciformis*, the most known species of the *Hypnea* genus, is a red marine macroalga species distributed throughout the world and is considered important as a source of dietary supplements due to its bioactive compounds such as sulfated polysaccharides (e.g., carrageenans) [10, 11]. Carrageenans from *H. musciformis* have been shown to inhibit breast cancer and neuroblastoma cell proliferation highlighting their anti-carcinogenic potential [10]. Moreover, administration of compounds from *H. musciformis* to a mouse model of Ehrlich ascites carcinoma resulted in the decrease of tumor size through increase of apoptotic molecules (e.g., p53, Bax, Caspase 3) [9, 12]. In addition, *H. musciformis* extract inhibited chemical-induced carcinogenesis in animal experimental models indicating a chemo-preventive activity [8].

Colon and liver cancers are among the malignancies that are considered appropriate for application of chemo-preventing approaches [13, 14]. Liver cancer is the fifth most frequent tumor-type worldwide and the third one in terms of mortality [7]. The conventional therapies used for liver cancer are ineffective, therefore alternative treatments, such as the use of natural compounds with anticancer properties, are needed [7]. For example, phytochemicals (e.g., quercetin), grape extracts and extracts from seaweeds such as *Ulva intestinalis* have been reported to inhibit liver cancer cell proliferation in vitro and in vivo through various mechanisms such as induction of apoptosis, cell cycle arrest, anti-angiogenesis and anti-metastasis [7, 13, 15]. Moreover, colon cancer is the second and third most common tumor worldwide in men and women, respectively [14]. Even though screening for colon cancer has decreased its frequency and mortality, chemoprevention may be used to decrease it even further or to impede its post-treatment recurrence [14]. Interestingly, *H. musciformis* extracts have been shown to inhibit HCT-116 colon cancer cells growth [12].

The dissection of molecular mechanisms accounting for the tumor-suppressing properties of natural products is of great importance for their establishment as chemo-preventive agents [7]. The use of integrated omic approaches can be a powerful research tool for investigating the molecular pathways that facilitate the anticancer activity of natural products [16, 17]. Functional genomics methods can reveal alterations in gene expression in response to treatment with natural compounds, which in turn help to elucidate the cell signaling pathways being responsible for their chemo-preventive effects in cancer cells [17]. Furthermore, the identification of specific molecular mechanisms through which anticancer activity is exerted can also facilitate their use for personalized prevention [18, 19].

In the present study, both transcriptomic and proteomic were deployed to investigate the molecular pathways and mechanisms through which *H. musciformis* extracts inhibited liver and colon cancer cell growth. It is the first time that functional genomic approaches were used for elucidating *H. musciformis* extract’s anticancer activity. These results can serve as a strong foundation for the potential development of *H. musciformis* extracts as food supplement possessing chemopreventive properties.

## Materials and methods

### *H. musciformis* collection and extract preparation

*H. musciformis* was collected from June to September 2020 from the Thermaikos Gulf (40°40′64.46″ N, 22°89′34.38″ E; Thessaloniki, Greece), Northern Aegean Sea, Mediterranean Sea. The identification of the *H. musciformis* was based on the following floral catalogs and studies [20–24], AlgaeBase, available online: https://www.algaebase.org/search/species, assessed on 13/11/2022]. Extract preparation from the red seaweed was performed as described in [25] with modifications. In brief, *H. musciformis* samples were grinded, soaked in 80% methanol solution (1:30 dried weight sample to solvent volume) and elaborated with sonication (UP400S Hielscher sonicator, Teltow, Germany) at 20 cycles and 70% amplitude for 20 min. Afterwards, the solution was left in a shaker incubator (Innova® 40, New Brunswick Scientific; St Albans, UK) at 25 °C and 150 rpm for 48 h. Then, the extract solution was filtered using Whatman filter paper (0.45 μm). The solvent was removed under reduced pressure by a rotary evaporator (IKA, Werke RV-06-ML; Staufen, Germany) at 30 °C and 150 rpm, followed by freeze drying (CoolsafeTM, Scanvac; Allerod, Denmark) for 24 h, to produce the *H. musciformis* extract (HME) in the form of powder.

### Cell culture

Human liver HepG2 cancer cell line was obtained from Dr. Anna-Maria Psarra (University of Thessaly, Larissa, Greece). Human intestinal Ls174 cells were a kind gift of Dr. Pantelis Hatzis (B.S.R.C “Alexander Fleming”, Vari, Greece) and have been described previously [26]. All cells were cultured in normal Dulbecco’s modified Eagle’s medium (DMEM; Gibko, UK), containing 10% (v/v) fetal bovine serum, 2 mM L-glutamine (Gibko, UK), 100 units/ml of penicillin, and 100 units/ml of streptomycin (Gibko, UK) in plastic disposable tissue culture flasks at 37 °C in 5% CO_2_.

### XTT cell proliferation assay

Inhibition of cell proliferation was assessed using the XTT assay kit (Roche, Germany), as described previously [27]. Briefly, 1 × 10^4^ cells were sub-cultured into a 96-well plate in DMEM medium. After 24-h incubation, the cells were treated with different concentrations of HME in serum-free DMEM medium for 24 h. 50 μL of XTT test solution, which was prepared by mixing 50 μL of XTT-labeling reagent with 1 μL of electron coupling reagent, was then added to each well. After 4 h of incubation, absorbance was measured at 450 nm and at 690 nm as a reference wavelength on a Perkin Elmer EnSpire Model 2300 Multilabel microplate reader (Waltham, MA, USA). Cells cultured in DMEM serum-free medium were used as a negative control. Also, the absorbance of each extract‘s concentration alone in DMEM serum-free medium and XTT test solution was tested at 450 nm. The absorbance values shown by the HME alone were subtracted from those derived from cancer cell treatment with extracts. Data were calculated as percentage of inhibition by the following formula:

1 $${\text{Inhibition}}\,\left( \% \right) = \left[ {\left( {{\text{O}}.{\text{D}}._{{{\text{control}}}} {-}{\text{O}}.{\text{D}}._{{{\text{sample}}}} } \right)/{\text{O}}.{\text{D}}._{{{\text{control}}}} } \right] \times 100$$

where O.D._control_ and O.D._sample_ indicated the optical density of the negative control and the tested substances, respectively. The concentration of HME causing 50% cellular proliferation inhibition (IC_50_) of cancer cells was calculated thereafter from the graph plotted percentage inhibition against extract concentration. All experiments were carried out at least on three separate occasions in triplicate.

### Cell culture treatments with HME

For transcriptome and proteome analysis, 3 × 10^5^ cancer cells were incubated without or with the HME at a concentration of 0.78 mg/ml for 24 h. This concentration was selected based on the concentration of IC_50_ of the extract in liver and intestinal cancer cells calculated by the cytotoxicity method XTT assay. The IC_50_ concentration for HME treatment was 0.82 mg/ml in HepG2 cells and 0.75 mg/ml in Ls174 cells. These concentrations were calculated using the XTT method from dose-dependent cell growth inhibition diagrams (see above).

### Assessment of cell apoptosis

The HME-induced cell death type, including both apoptosis and necrosis, was assessed by flow cytometry using the dual staining Annexin V-Phycoerythrin (Annexin V-PE) and 7-Amino-actinomycin D (7-AAD) apoptosis detection kit (BD Pharmingen™, San Diego, CA, USA) according to the manufacturer’s instructions. In brief, HepG2 or Ls174 cells were cultured in 6-well plates (0.3 × 10^6^ cells/well) in DMEM containing 10% FBS for 24 h. Then, cells were treated with 0.78 mg/ml (IC_50_) of HME in DMEM without FBS for 0.5, 1, 2, 4, 8, 16 and 24 h. Afterwards, cells were harvested by 0.25% trypsin and washed twice with cold phosphate buffered saline solution (PBS; pH 7.4), and then they were resuspended in 100 μl of 1 × binding buffer at a concentration of 1 × 10^6^ cells/ml. Cells were stained by adding 5 μl of Annexin V-PE and 5 μl of 7-AAD and incubated for 15 min at RT in the dark. After incubation, 400 μl of 1Χ binding buffer were added to each sample and flow cytometry (FACSCalibur, BD Biosciences, NJ, USA) was performed within 1 h to detect live (Annexin V-PE−/7-AAD−), early apoptotic (Annexin V-PE +/7-AAD−), late apoptotic (Annexin V-PE+/7-AAD +) and necrotic (Annexin V-PE−/7-AAD+) cells. The analysis of the results was made using CellQuest software (BD Biosciences, NJ, USA).

### Assessment of mitochondrial membrane potential

Mitochondrial membrane potential (ΔΨm) was measured by tetramethylrhodamine ethyl ester (TMRE) (MitoStatus TMRE, BD Pharmingen™, San Diego, CA, USA) according to the manufacturer’s instructions. Briefly, HepG2 or Ls174 cells (1 × 10^6^ cells/well in 6-well plates) were treated with 0.78 mg/ml (IC_50_) of HME in DMEM without FBS for 0.5, 1, 2, 4, 8, 16 and 24 h. After culture medium removal, cells were washed with PBS and stained with 100 nM of TMRE dye dissolved in stain buffer (#554,656, BD Pharmingen™, San Diego, CA, USA) for 30 min at 37 °C in the dark. Afterwards, cells were harvested by 0.25% trypsin, washed twice with stain buffer, and finally resuspended in 100 μl of stain buffer at a concentration of 1 × 10^6^ cells/ml. Then, the fluorescence intensity of cells was measured with flow cytometry (FACSCalibur, BD Biosciences, NJ, USA) and analyzed using CellQuest software (BD Biosciences, NJ, USA).

### Total RNA isolation and real-time quantitative PCR

For transcriptome analysis, total nucleic acid (TNA) isolation from the two cell lines (Ls174, HepG2) with (experimental condition) and without (control) addition of HME was performed with Trizol (MRC, #TR-119) according to manufacturer's instructions. Both experimental conditions and controls were performed in biological triplicates. The final TNA precipitates were redissolved at 50 ul ddH20, and their concentration was determined spectrophotometrically (Q3000, Quawell CA, USA, www.quawell.com). After extraction all samples were stored at − 80 °C until NGS library construction. 10 ug of Total Nucleic Acid (TNA) were DNase-treated (#M6101 RQ DNase I Promega) via incubation at 37 °C for 1 h, followed by phenol/chloroform extraction and ethanol precipitation. 1 ug of DNAse-treated RNA was used for first-strand cDNA synthesis (#28,025,013 MMLV reverse transcriptase, Invitrogen) via incubation at 37 °C for 2 h, primed with random hexamers according to manufacturer’s recommendation. RT-qPCR reactions were performed with KAPA SYBRGREEN qPCR mix (Sigma # KR0389_S) as previously described [28]. All primers used for RT-qPCR are shown in Additional file 1: Table S1.

### Chromatin immunoprecipitation (ChIP)

ChIP assay was performed as previously described [26]. In brief, 4 × 10^6^ cells from each cell line with and without HME treatment were used per IP. The chromatin was incubated with 4 μg of anti-p53 (Santa Cruz, #sc-126) or anti-P300 (Santa Cruz, #sc-48343). Immunoprecipitated DNA was de-crosslinked, extracted with phenol/chloroform and precipitated with ethanol prior to RT-qPCR analysis. All primers used for ChIP-qPCR analysis are shown in Additional file 1: Table S2.

### Western blotting (WB)

The total protein of immunoprecipitated chromatin along with input chromatin per cell line and condition was obtained using 2 × SDS lysis buffer (100 mM Tris pH 6.8, 4% SDS, 10% glycerol, 2% 2-Mercaptoethanol) and then separated by SDS-PAGE. The protein was transferred onto nitrocellulose membrane (#NBA085C001EA, Protran nitrocellulose, PerkinElmer) and incubated with primary antibodies overnight after blocking for 1 h at RT in the blocking solution (5% skim milk in TBST). Subsequently, TBST was used to wash membranes four times, adding the secondary antibody at the dilution of 1: 10,000 in TBST to incubate for 1 h. Finally, the ECL chemiluminescence kit (#RPN2109 Amersham) was utilized to visualize proteins after washing four times. The antibodies used were the same as for ChIP.

### 3′ end RNA sequencing

A total of 10 ug of TNA from each sample were sent to the Genomic Analysis Unit of the B.S.R.C “Alexander Fleming” (https://www.fleming.gr/facilities/genomics) for NGS library construction sequencing. The RNA quality and quantity of each sample was estimated with the Bioanalyzer (Agilent Technologies) using the reagents and the Agilent RNA 6000 Nano Kit protocol. Samples with concentrations that exceeded 500 ng/μl were diluted accordingly to an average concentration of 150 ng/μl. Sample preparation and library construction were performed according to the protocol and reagents of the 3′ mRNA-Seq Library Prep Kit for Ion Torrent (QuantSeq-LEXOGEN™ Vienna, Austria https://www.lexogen.com/quantseq-ion-torrent/), following the manufacturer's instructions. Briefly, 300 ng per 5 μl of RNA from each sample were used to perform the first strand synthesis. Any remaining RNA was removed, and 2nd strand synthesis was initiated with a random primer, containing sequences of Ion Torrent-compatible links at the 5′ end. At this point, bar codes were inserted. Second strand synthesis was followed by a cleaning step that is based on magnetic beads and the resulting library was amplified for 13–18 cycles and subjected to a second clean-up. The quality and quantity of each library was then evaluated with the Bioanalyzer using the reagents and the DNA Protocol High Sensitivity Kit (Agilent Technologies). The quantified libraries were assembled in 12plex at a final concentration of 7 pM. The library pool was processed in the Ion Proton One Touch system, in which the libraries were enriched with the Ion PI™ Hi-Q™ OT2 200 kit (ThermoFisher Scientific) and sequenced with the Ion PI™ Hi-Q™ 200 kit (ThermoFisher Scientific) on an Ion proton PI™ V2 chip (ThermoFisher Scientific) according to commercially available protocols [29].

### RNA-seq bioinformatic and statistical analysis

The raw sequencing data (FASTQ files) were subjected to a quality control using the FASTQC package (https://www.bioinformatics.babraham.ac.uk/projects/fastqc/) and subsequently were aligned against the human genome (hg19 assembly) using Hisat2 (http://daehwankimlab.github.io/hisat2/) with default settings, using Illumina iGenomes (http://cufflinks.cbcb.umd.edu/igenomes.html). The resulting BAM files were statistically analyzed using the Bioconductor package MetaseqR2 [30]. Genes showing a binomial test *p*-value of less than 0.05 and a deviation range (for each contrast) greater than 0.58 or less than − 0.58 on the log2 scale (corresponding to 1.5 times up or down, respectively) were considered as being differentially expressed. Volcano and Deregulogram analyses were visualized using the ggplot2 package (https://ggplot2.tidyverse.org/) in R. Venn analyses were performed with the Venny program (https://bioinfogp.cnb.csic.es/tools/venny/). Gene Ontology analyses for the differentially expressed genes were performed with Gencodis (https://genecodis.genyo.es/) [31] and visualized with ggplot2 in R. Network analysis was performed in STRING (https://string-db.org/) with default parameters. Statistical analysis of gene overlaps was performed by hypergeometric control tests in R.

### Protein isolation

For the proteome analysis, total protein isolation was performed for both cell lines as described above for the transcriptome analysis. In brief, cells were trypsinized, centrifuged at 1000 rpm for 3 min at 4 °C and the cell pellet was resuspended at 40 ul of lysis buffer (4% SDS, 0.1 M DTT, 0.1 M Tris pH 7.4). Total protein concentration was determined spectrophotometrically (Q3000, Quawell CA, USA, www.quawell.com). After isolation all samples were stored at − 80 °C until LC–MS/MS analysis.

### Bottom-up proteomic sample preparation using the Sp3 mediated protein digestion protocol

The lysed samples were processed according to the sensitive Sp3 protocol [32]. The cysteine residues were reduced in 100 mM DTT and alkylated in 200 mM iodoacetamide (Acros Organics). 20 ug of beads (1:1 mixture of hydrophilic and hydrophobic SeraMag carboxylate-modified beads, GE Life Sciences) were added to each sample in 50% ethanol. Protein clean-up was performed on a magnetic rack. The beads were washed two times with 80% ethanol and once with 100% acetonitrile (Fisher Chemical). The captured on-beads proteins were digested overnight at 37 °C under vigorous shaking (1200 rpm, Eppendorf Thermomixer) with 0.5 ug Trypsin/LysC (MS grade, Promega) prepared in 25 mM ammonium bicarbonate. Next day, the supernatants were collected, and the peptides were purified using a modified Sp3 clean up protocol and finally solubilized in the mobile phase A (0.1% Formic acid in water), sonicated and the peptide concentration was determined through absorbance at 280-nm measurement using a nanodrop instrument.

### LC–MS/MS analysis

Samples were run on a liquid chromatography tandem mass spectrometry (LC–MS/MS) setup consisting of a Dionex Ultimate 3000 nano RSLC online with a Thermo Q Exactive HF-X Orbitrap mass spectrometer. Peptidic samples were directly injected and separated on an 25-cm-long analytical C18 column (PepSep, 1.9μm3 beads, 75 µm ID) using an one-hour long run, starting with a gradient of 7% Buffer B (0.1% Formic acid in 80% Acetonitrile) to 35% for 40 min and followed by an increase to 45% in 5 min and a second increase to 99% in 0.5 min and then kept constant for equilibration for 14.5 min. A full MS was acquired in profile mode using a Q Exactive HF-X Hybrid Quadropole-Orbitrap mass spectrometer, operating in the scan range of 375–1400 m/z using 120 K resolving power with an AGC of 3 × 106 and max IT of 60 ms followed by data independent analysis using 8 Th windows (39 loop counts) with 15 K resolving power with an AGC of 3 × 105 and max IT of 22 ms and a normalized collision energy (NCE) of 26.

### Data analysis

Orbitrap raw data was analyzed in DIA-NN 1.8 (Data-Independent Acquisition by Neural Networks, [33]) through searching against the reviewed Human Uniprot database (retrieved 4/21) in the library free mode of the software, allowing up to two tryptic missed cleavages. A spectral library was created from the DIA runs and used to reanalyze them. DIA-NN default settings have been used with oxidation of methionine residues and acetylation of the protein N-termini set as variable modifications and carbamidomethylation of cysteine residues as fixed modification. N-terminal methionine excision was also enabled. The match between runs (MBR) feature was used for all analyses and the output (precursor) was filtered at 0.01 FDR and finally the protein inference was performed on the level of genes using only proteotypic peptides. The proteomics data were processed in Perseus 1.6.15.0 [34]. Values were log(2) transformed, a threshold of 70% of valid values in at least one group was applied and the missing values were replaced from normal distribution. The generated results were processed statistically in the Perseus software (1.6.15.0, [34]) with Student’s t-test and permutation-based FDR calculation and visualized in R with ggplot2.

### Statistical analyses

All experiments were repeated at least three times and the experimental data were expressed as the mean ± SD. One-way ANOVA was performed with SigmaPlot to compare differences between groups. *p < 0.05 indicates statistical significance at significant level 0.05.

## Results

### HME inhibits liver and intestinal cancer cell proliferation

**Fig. 1:**
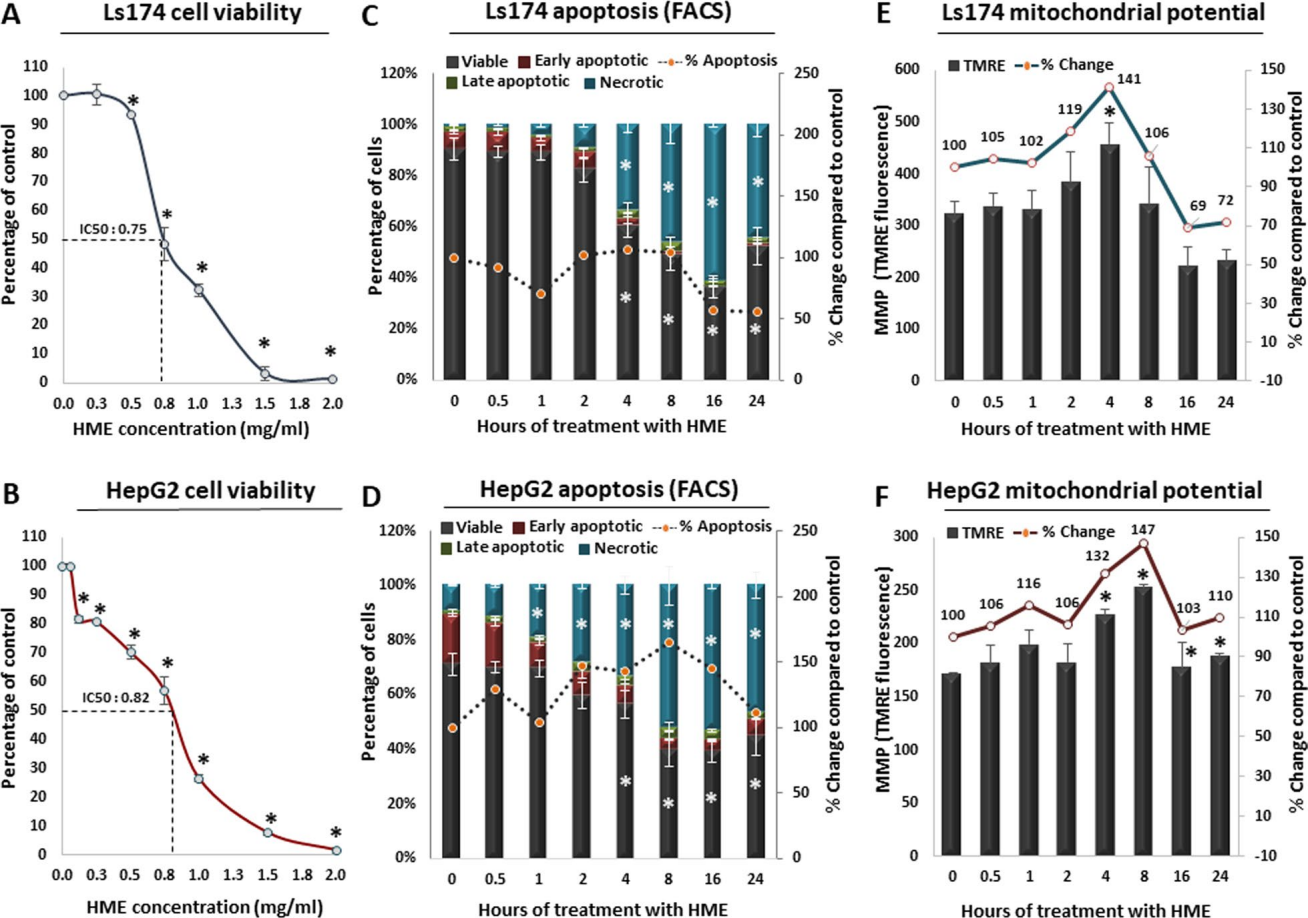
Cytotoxicity and cell death measurements after *Hypnea musciformis* extract (HME) treatment. Dose-dependent inhibition of HME against **A** Ls174 intestinal and **B** HepG2 liver cancer cell proliferation. IC_50_ values are indicated for each cell line. Cytotoxicity was evaluated using XTT assay. Each point represents mean value ± SD. **C** Flow cytometry analysis of Ls174 cells stained with Annexin V-PE and 7-AAD, highlighting the percentage of viable, early and late apoptotic and necrotic cells after treatment with HME for 0.5, 1, 2 4, 8, 16 and 24 h, respectively. Each bar represents mean value ± SD. Dashed line demonstrates the change of apoptotic cells as percentage compared to the untreated control. **D** Same as in **C** but for HepG2 cells. **E** Effect of HME on mitochondrial membrane potential (MMP) in Ls174 cells. Each bar represents mean value ± SD. Line demonstrates the change of MMP as percentage compared to the untreated control. **F** Same as in **E** but for HepG2 cells. Asterisk marks statistical significance (*p* < 0.05) in all panels

The ability of HME to inhibit proliferation of Ls174 intestinal and HepG2 liver cancer cells was examined after treatment for 24 h with increasing extract concentrations using the XTT assay (Fig. 1; Additional file 1: Table S3). The results showed that HME treatment significantly inhibited both Ls174 (Fig. 1A) and HepG2 (Fig. 1B) cell proliferation in a dose-dependent manner. The HME’s concentration, in which cell growth was inhibited by 50%, was calculated at 0.75 and 0.82 mg/ml for Ls174 and HepG2 cells, respectively. Importantly, although the IC_50_ for Ls174 cells is slightly lower compared to the HepG2 cells, the latter exhibit a sharp reduction of cell viability even at low HME concentrations compared to the Ls174 counterpart, overall supporting a stronger cytotoxic response of the extract in liver cancer cells.

### Effect of HME on cancer cell apoptosis and necrosis

To determine whether treatment of cancer cells with HME triggers a more general anti-tumoral response apart from the observed cytotoxic effects, the percentage of apoptotic cells were assessed with Annexin V-Phycoerythrin (Annexin V-PE) and 7-Amino-Actinomycin (7-AAD) double staining (Fig. 1; Additional file 2: Fig. S1; Additional file 1: Table S3) and subsequently coupled to flow cytometry following treatment with HME for 0.5, 1, 2, 4, 8, 16 and 24 h. The results showed that the viability of Ls174 cells was significantly reduced to 60.7, 48.8, 36.5 and 52.5% after 4, 8, 16 and 24 h treatment, respectively (Fig. 1C; Additional file 2: Fig. S1A; Additional file 1: Table S3). In parallel, a significant increase in Ls174 late apoptotic cells was observed after 8, 16 and 24 h of HME treatment compared to control (Fig. 1C; Additional file 2: Fig. S1A; Additional file 1: Table S3). Importantly, the percentage of Ls174 necrotic cells was significantly increased by 32.3, 44.4, 59.8 and 43.5 after 4, 8, 16 and 24 h treatment, respectively, compared to control (Fig. 1C; Additional file 2: Fig. S1A; Additional file 1: Table S3).

HME treatment of HepG2 cells significantly decreased the percentage of viable cells to 56.6%, 40.2%, 39.4% and 45.2% after 4, 8, 16 and 24 h treatment, respectively (Fig. 1D; Additional file 2: Fig. S1B; Additional file 1: Table S3). In addition, the percentage of HepG2 late apoptotic cells was significantly increased by 2.5-fold after only 2-h treatment with HME compared to control and continued with the same average fold change between 4 and 24 h (Fig. 1D; Additional file 2: Fig. S1B; Additional file 1: Table S3). Consequently, HME treatment rapidly and significantly increased the percentage of HepG2 necrotic cells by 18.9%, 23.9%, 42.5%, 43.6% and 36.8%, after 2, 4, 8, 16 and 24 h treatment, respectively, compared to control (Fig. 1D; Additional file 2: Fig. S1B; Additional file 1: Table S3). In conclusion, according to these results, HME treatment triggers an apoptotic response both to Ls174 and HepG2 cancer cells; however, the kinetics of this effect are faster in HepG2, resulting into an overall stronger cumulative impact on liver cancer cell survival.

### Effect of HME on mitochondrial membrane potential (MMP) in intestinal and liver cancer cells

Next, we measured the potential of the mitochondrial membrane to address whether the observed effects of HME treatment on cancer cell survival can be explained in part through impairment of mitochondrial function. According to these results, treatment of Ls174 cells with HME caused a significant hyperpolarization by 41% after 4 h treatment compared to control, followed by a statistically significant sharp decrease of MMP by 31% and 28%, after 16 and 24 h, respectively, compared to control (Fig. 1E; Additional file 2: Fig. S1C; Additional file 1: Table S3).

In HepG2 cells, HME caused a significant increase of 32% and 47% in MMP after treatment for both 4 and 8 h, respectively, compared to control (Fig. 1F; Additional file 2: Fig. S1D; Additional file 1: Table S3). However, unlike Ls174 cells we did not observe a decline of MMP in these cells at any time point compared to control. To conclude, based on the MMP results, HME treatment significantly impairs mitochondrial function both in colon and liver cancer cells.

### Transcriptome analysis of cancer cells treated with HME

**Fig. 2:**
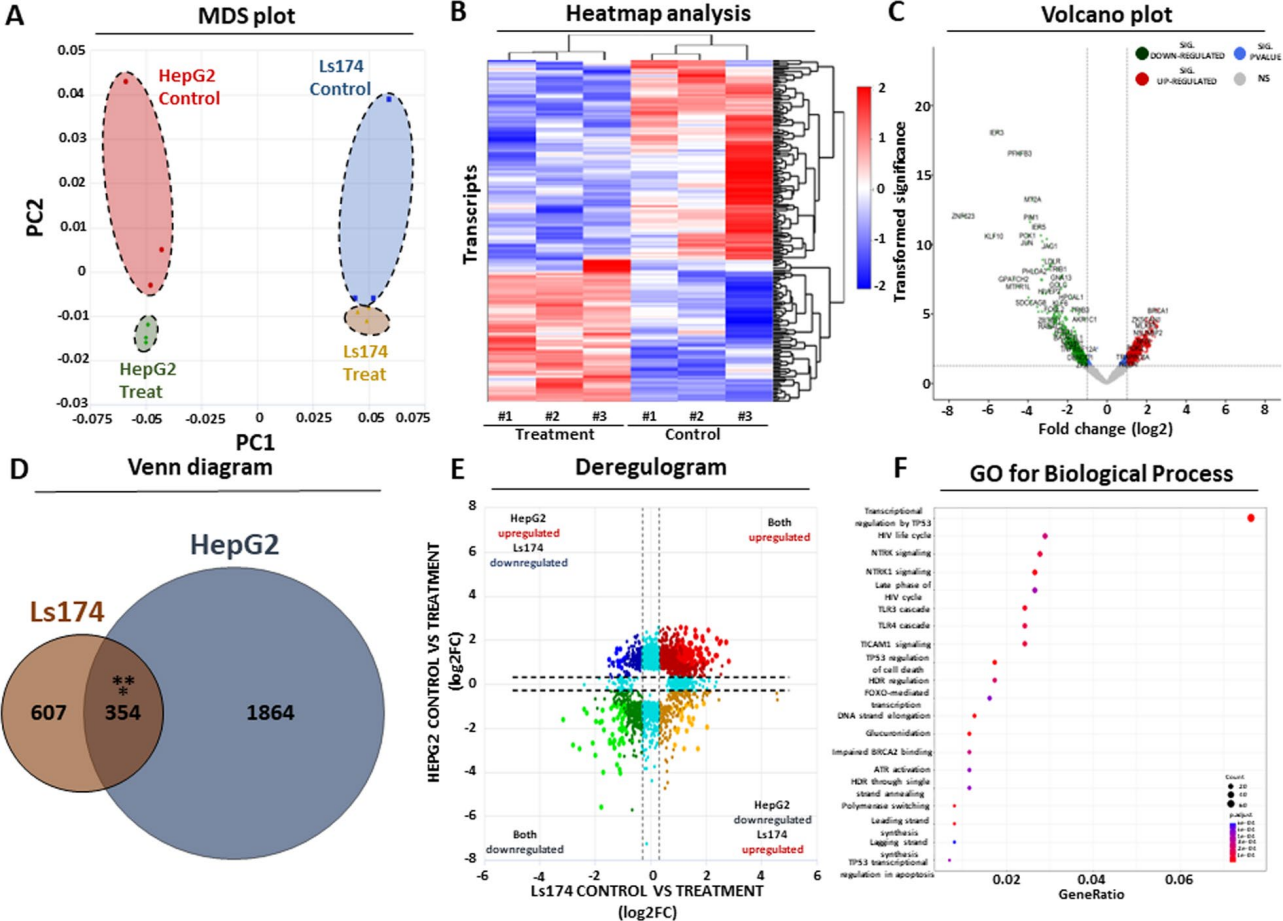
Transcriptome analysis following HME treatment. **A** MDS plot analysis highlighting the two primary sources of transcriptional variability in HepG2 and Ls174 cells following seaweed extract treatment. Each dot corresponds to a separate biological replicate. PC: principal component. **B** Heatmap analysis summarizing the transcriptional changes in HepG2 cells with and without treatment. Gene expression was log2 transformed and shown as normalized z-score. **C** Volcano plot analysis demonstrating the statistically significant differentially expressed (DE) genes following treatment of HepG2 cells with seaweed extract. *Y*-axis represents *p*-value after − log10 transformation, *x*-axis corresponds to log2 transformed fold change. **D** Venn diagram showing the integration of HepG2 and Ls174 statistically significant DE genes in response to seaweed treatment. Asterisks mark statistical significance based on a hypergeometric test. **E** Deregulogram analysis highlighting the unidirectional expression changes of the commonly affected transcripts in Ls174 and HepG2. Both axes correspond to log2 fold change of treated vs untreated cells. **F** Dot plot summarizing gene ontology results of biological function for the statistically significant DE genes. Dot size corresponds to number of DE genes and colour corresponds to statistical significance for each cellular function

We subsequently subjected both HME treated and untreated HepG2 and Ls174 cells into transcriptome and proteome analysis with the aim of dissecting the observed phenotype at the molecular level. Based on the transcriptome results, incubation with the seaweed extract led to profound changes of gene expression, effectively separating treated from untreated cells as observed in the MDS plot (Fig. 2A). Although one of the control biological replicates deviated from the rest in both cell lines, the separation of treated versus untreated cells was more observable in the HepG2 liver cancer cell line compared to the Ls174 colon cancer cells (Fig. 2A). We did not observe any expression bias for a specific transcript biotype or chromosome, suggesting that the seaweed extract affects gene expression from all annotated classes of human transcripts across the genome (data not shown).

Next, we focused on detecting the differentially expressed transcripts between treated and untreated cells for both cell lines. Heatmap analysis effectively separated treated from untreated HepG2 (Fig. 2B) or Ls174 (Additional file 3: Fig. S2A) cells, confirming the observed impact of the seaweed extract on gene expression of both cancer cell lines (Fig. 2A). Volcano plot analysis revealed 2,218 statistically significant differentially expressed (DE) genes in HepG2 cells (Fig. 2C), out of a total number of 27,326 annotated genes that remained after filtering of low/no expressed genes (DE percentage: 8.11%). These DE genes were further subdivided into 1,360 statistically significant upregulated (treated vs untreated fold change ≥ 0.5 and *p* value < 0.05) and 858 significantly downregulated genes (treated vs untreated fold change ≤  − 0.5 and *p* value < 0.05), corresponding to 61.3% and 38.7% of the total DE gene for this cell line, respectively. In Ls174 cells, we detected 961 DE genes, corresponding to 3.51% of total annotated genes after filtering (Additional file 3: Fig. S2B). Out of them, 717 were statistically significantly upregulated (treated vs untreated fold change ≥ 0.5 and *p* value < 0.05) while 244 were statistically significantly downregulated (treated vs untreated fold change ≤  − 0.5 and *p* value < 0.05), corresponding to 74.6% and 25.4% of all DE genes for this cell line, respectively. In conclusion, a strong transcriptional response is observed both in the HepG2 and the Ls174 cells after HME treatment.

### Detection of mutually affected DE transcripts

Having detected hundreds of DE genes in response to seaweed extract treatment in both cancer cell lines, we next entertained the possibility of shared transcriptional responses between the two cell types. Venn diagram analysis revealed a small yet statistically significant overlap of 354 DE genes (11% of total DE genes for both cell lines) that were common between HepG2 and Ls174 cells (Fig. 2D). To determine whether these commonly affected transcripts also change their expression in a unidirectional manner, we visualized the RNA-seq data in the form of a deregulogram that detects patterns of significant DE genes between two comparisons (Fig. 2E). According to these results, HepG2 and Ls174 cells share a small number of unidirectionally affected DE genes; however, most transcriptional perturbations in both cell types are expressed in the form of unique transcriptional responses to HME treatment.

### Gene ontology analysis of differentially expressed genes

To link the observed transcriptional changes of HepG2 and Ls174 cells to biological processes, we independently subjected the detected DE transcripts of each cell line to a gene ontology (GO) analysis, with the aim of detecting biological functions that are significantly enriched in response to the observed transcriptional responses for each cell line. GO analysis of the DE transcripts in the HepG2 background revealed a strong transcriptional footprint of p53-mediated regulation (Fig. 2F). The observed p53-mediated response was further specialized into regulation of targets that are associated with programmed cell death as well as ATR- and BRCA-associated DNA damage response and p53-mediated regulation of apoptosis. Interestingly, GO analysis of the Ls174 DE transcripts deviated from the HepG2 results, revealing enrichment of cellular functions that are more associated with metabolic effects, including metabolism of glycoproteins, phospholipids and cholesterol (Additional file 3: Fig. S2C). In conclusion, GO analysis revealed a separate response of these cellular backgrounds to the extract treatment, which predominantly associates with a strong regulatory footprint of p53 in HepG2 cells and a strong alteration of metabolic properties in the Ls174 background. These results were also confirmed with KEGG pathway analysis (Additional file 1: Table S4).

### Proteome analysis of cancer cells treated with HME

**Fig. 3:**
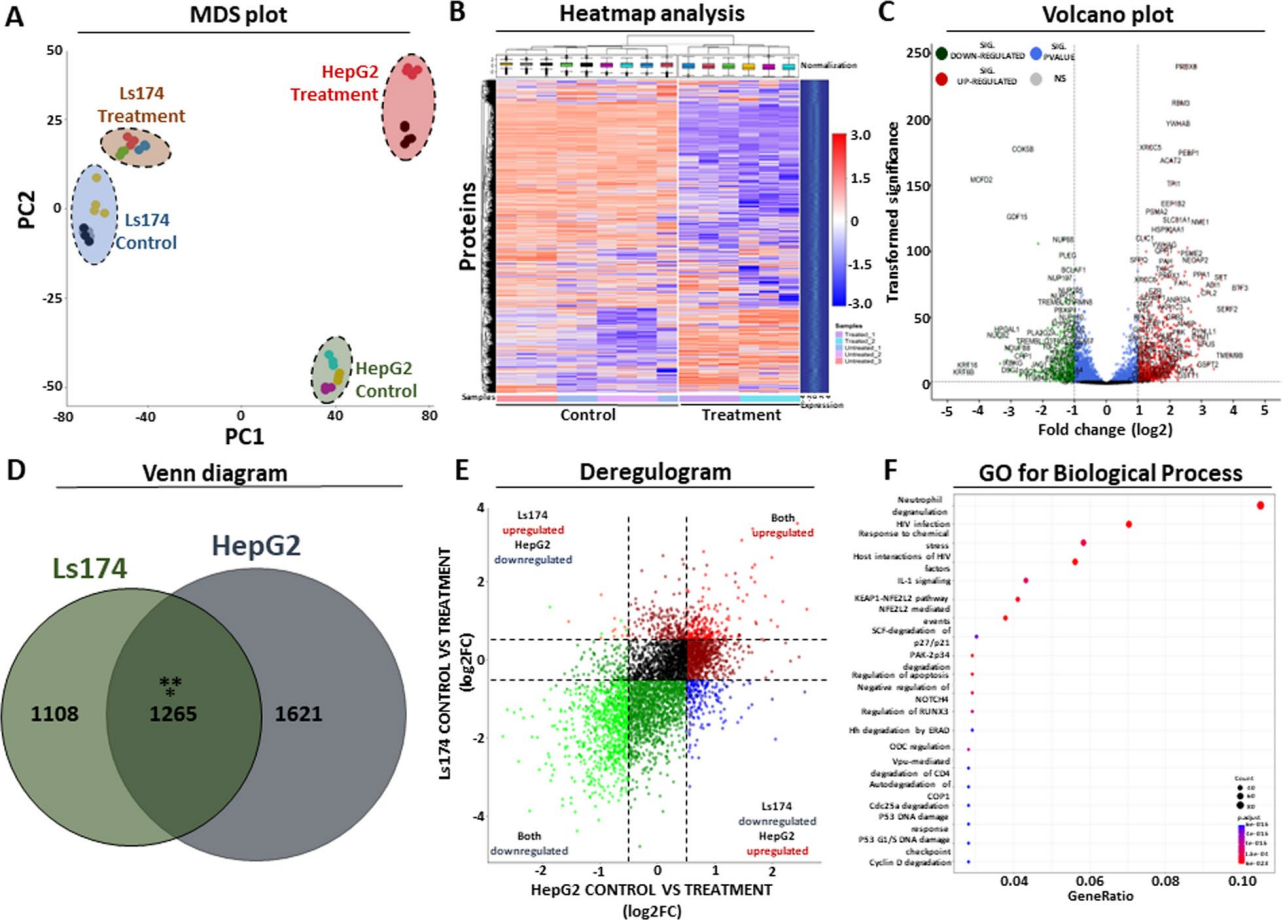
Proteome analysis after treatment with HME. **A** MDS plot analysis of LC/MS–MS results in HepG2 and Ls174 cells after treatment with seaweed extract. Each dot corresponds to a separate technical replicate, colours summarize different biological replicates. PC: principal component. **B** Heatmap plot illustrating protein alterations in HepG2 cells with and without treatment. Protein levels were log2 transformed and shown as normalized z-score. **C** Volcano plot pinpointing the statistically significant differentially expressed proteins in HepG2 cells after treatment with the seaweed extract. *Y*-axis represents *p*-value after − log10 transformation, *x*-axis corresponds to log2 transformed fold change in protein levels. **D** Venn diagram showing the integration of HepG2 and Ls174 statistically significant protein alterations in response to seaweed treatment. Asterisks mark statistical significance based on a hypergeometric test. **E** Deregulogram plot showing the unidirectional change of the commonly affected proteins in Ls174 and HepG2. Both axes correspond to log2 fold change of treated vs untreated cells. **F** Dot plot summarizing gene ontology results of biological function for the statistically significant alterations in protein levels. Dot size corresponds to number of DE proteins and colour corresponds to statistical significance for each cellular function

We also characterized the effect that the HME treatment exhibits at the proteome of the two cancer cells. Consistent with the transcriptome observations, LS-MS/MS analysis confirmed that the proteome of treated cells is altered compared to the untreated control and that the HepG2 cells exhibit a stronger pattern of deregulation over the Ls174 background (Fig. 3A). The observed variability between the biological and technical replicates of the analysis was significantly smaller than the variability between treated and control cells, inclining toward the existence of many significantly deregulated proteins. In agreement with the observations of the transcriptome MDS analysis, treatment of both cell lines with the extract served as the second strongest source of variability on the dataset (following the variability that is attributed to the different cellular content), confirming its strong regulatory effect on the proteome of both cell lines. Heatmap analysis of the HepG2 (Fig. 3B) and the Ls174 (Additional file 3: Fig. S2D) proteome confirmed the results of the MDS analysis, revealing strong alterations for hundreds of proteins that effectively separate treated from control cells, while maintaining co-clustering of biological and technical replicates in all conditions.

Statistical analysis of the LC–MS/MS results revealed 3,506 statistically significant altered proteins in HepG2 cells, out of 7,049 that were detected in total (DE 49.7%). If fold change (see above) is also considered as a filtering criterium, then the total number of statistically significant DE proteins is reduced to 2,886, corresponding to 40.9% of the total for this background (Fig. 3C). Based on the same thresholds of the transcriptome analysis (see above), the HepG2 DE proteins are further subdivided into 1,410 significantly up-regulated proteins and 1,476 significantly down-regulated proteins that correspond to 48.8% and 51.1% of total DEs for this cell line, respectively. Similarly, the Ls174 cells yielded 3,627 significantly altered proteins corresponding to 51.4% of the total that were detected. If fold change is also considered, then the total number of significantly DE proteins is reduced to 2,373 or 33.6% of the total (Additional file 3: Fig. S2E). These are further analyzed to 1,051 significantly up-regulated and 1,322 significantly down-regulated proteins, representing 44.3% and 55.7% of total DE proteins for this cell line. In conclusion, these results support the existence of extensive changes in the proteome of both cell lines in response to incubation with HME. In addition, a pronounced pattern of altered expression is detected both in HepG2 and Ls174 cells, which is consistent with the observed results of the preceding transcriptomic analysis. However, we did not observe a significant correlation between the transcriptome and the proteome responses in both cell lines (*R*^2^ =  ~ 0.1). Finally, it is worth noting that in the proteome of HepG2 cells, a balance between up- and down-regulating proteins exists, in contrast to Ls174 where downregulated proteins slightly predominate.

### Detection of commonly deregulated proteins

To pinpoint common patterns in the proteomic responses of the two cell lines following incubation with HME, a combined analysis of the differentially expressed proteins from both cell lines was performed in the form of a Venn diagram (Fig. 3D). A statistically significant overlap of 1,265 DE proteins was observed between the two cell lines. Visualization of the two affected proteomes in the form of a deregulogram confirmed that the alterations of the overlapping proteins are unidirectional (*R*^2^ = 0.34, Fig. 3E), in agreement with the observations of the transcriptome analysis. In conclusion, statistical analysis revealed significant changes in the proteome after treating both cell lines with the seaweed extract. These responses are generally balanced, yet a stronger upregulated footprint in the proteome of HepG2 cells both qualitatively and quantitatively exists. Finally, a statistically significant overlap of unidirectional DE proteins is detected between the two cell lines, although again both proteomes demonstrate unique responses to HME treatment.

### Gene ontology analysis for differentially expressed proteins

GO analysis of DE proteins in HepG2 cells (Fig. 3F) revealed again a p53-mediated signature in agreement with the observations from the transcriptome analysis. This footprint was further specialized in p53-related DNA damage and cell cycle checkpoint responses including cyclin D suppression (Fig. 3F). Interestingly, GO analysis for the Ls174 DE proteins deviated from the observed HepG2 results, revealing enrichment of cellular functions related to the metabolism of nucleic acids, terpenoids, cholesterol as well as proteins that are related to the organization of the mitotic spindle (Additional file 3: Fig. S2F). In conclusion, GO analysis of DE proteins in Ls174 cells revealed a transcriptional footprint associated with cellular metabolism, while in HepG2 cells an overall response involving the p53 transcription factor is observed, leading to dysregulation of genes associated with cell damage repair and death as well as cell cycle checkpoints. These observations are consistent with the GO results of the transcriptome analysis, in which the footprint of p53-mediated regulation is mainly observed in HepG2 cells, while dysregulation of the Ls174 proteome is more related to metabolic responses.

### Validation of a p53-mediated response in HepG2 cells

**Fig. 4:**
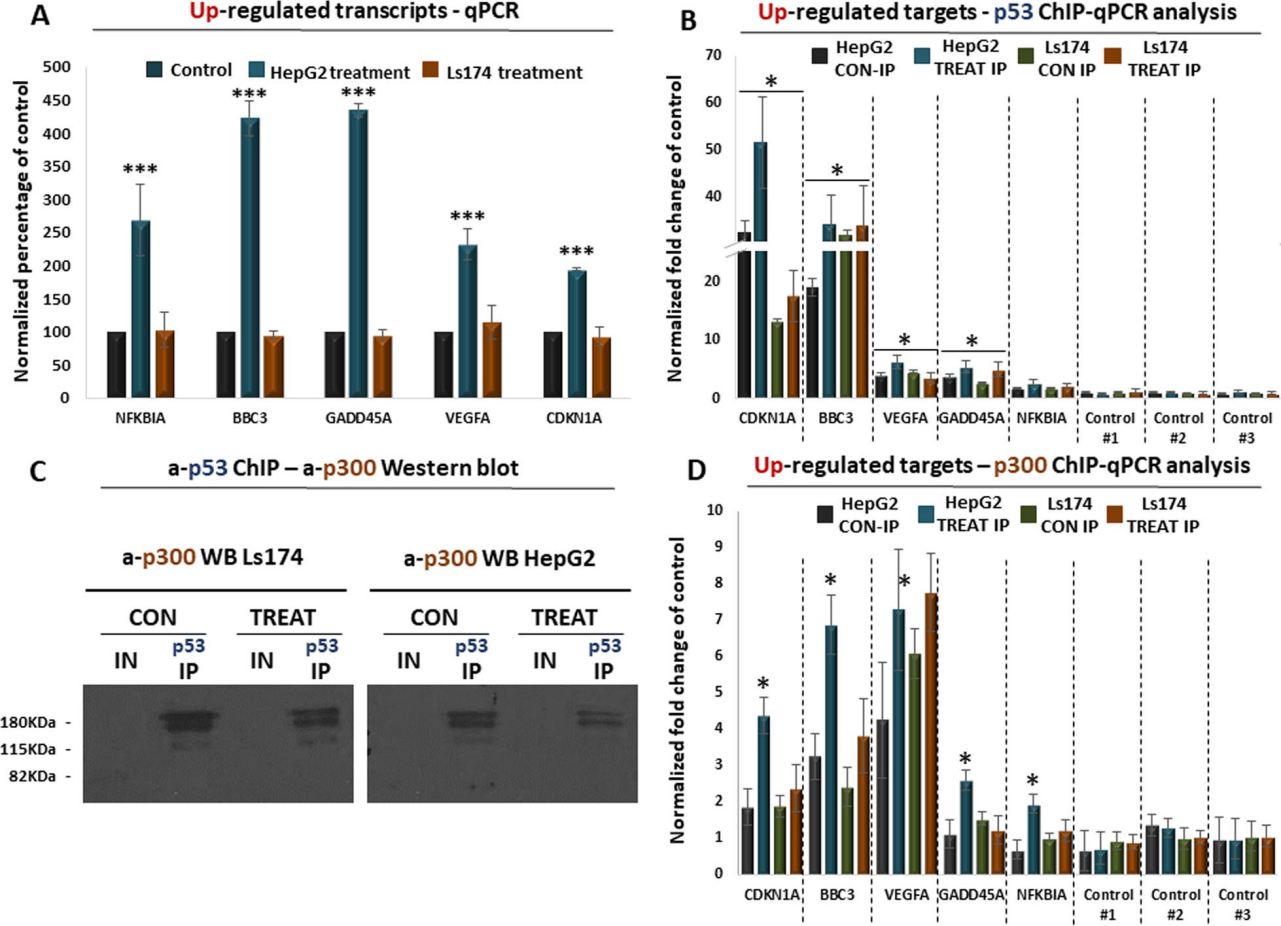
RT-qPCR and ChIP-qPCR analysis of selected target genes. **A** RT-qPCR analysis of known p53 target genes in HepG2 and Ls174 cells following treatment to seaweed extract. Control corresponds to untreated cells. Asterisks mark statistical significance based on ANOVA. **B** ChIP-qPCR for the HepG2-upregulated target genes, following immunoprecipitation with a-p53 in treated and untreated HepG2 and Ls174 cells. *Y*-axis corresponds to input normalized fold change compared to control loci. Asterisks mark statistical enrichment over control loci based on ANOVA. **C** ChIP-Western showing co-immunoprecipitation of the p300 protein with p53. **D** ChIP-qPCR for the HepG2 upregulated target genes, following immunoprecipitation with a-p300 in treated and untreated HepG2 and Ls174 cells. *Y*-axis corresponds to input normalized fold change compared to control loci. Asterisks mark statistical enrichment over control loci based on ANOVA

Following the observations from the transcriptome and proteome analysis, we subsequently focused on the observed p53-mediated response in HepG2 cells. qPCR analysis confirmed that several common p53 target genes are significantly upregulated in treated HepG2 cells compared to the untreated control, a trend that was not observed in Ls174 cells (Fig. 4A; Additional file 1: Table S5). These target genes are associated with cell cycle arrest, apoptosis and DNA damage repair, which are among the enriched cellular responses from the GO analysis (Figs. 2F, 3F). We therefore hypothesized that the p53 protein significantly interacts with the chromatin of the corresponding target loci. Indeed, ChIP-qPCR analysis confirmed a significant enrichment of p53 in the chromatin of several upregulated target genes compared to the chromatin of control loci (Fig. 4B; Additional file 1: Table S6). Interestingly, we also observed that p53 chromatin binding was further increased in treated compared to untreated HepG2 cells, a trend that was not observed in Ls174 cells for the same target genes (Fig. 4B). In conclusion, we have confirmed the p53-mediated transcriptional response in HepG2 cells and linked it to increased interactions of the p53 protein with the chromatin of the target genes, the molecular function of which explains part of the observed phenotypes.

**Fig. 5:**
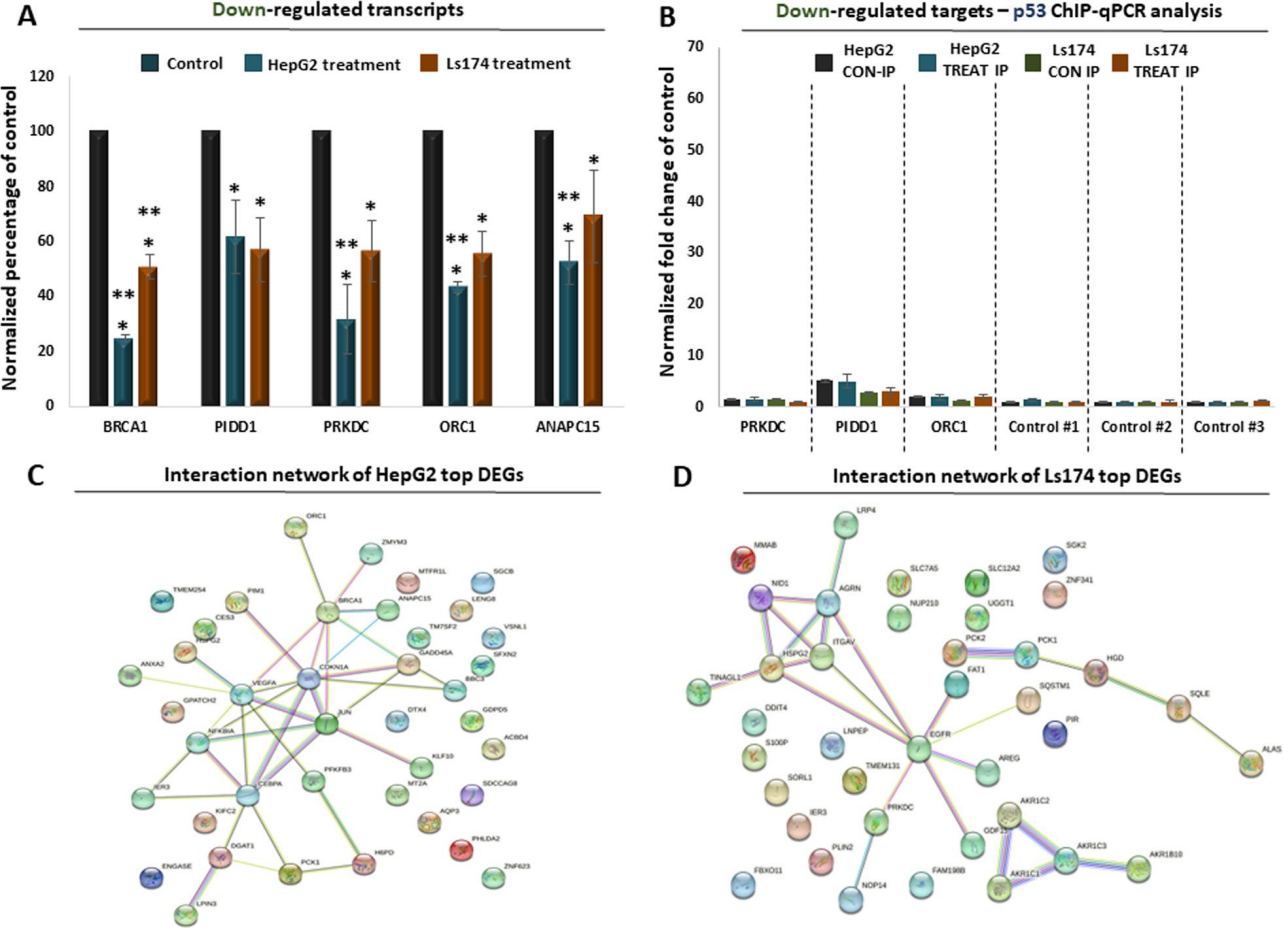
RT- and ChIP-qPCR analysis of downregulated target genes. **A** RT-qPCR analysis of selected downregulated genes in Ls174 and HepG2 cells following treatment with seaweed extracts. *Y*-axis shows fold change of expression in treated cell levels compared to control that corresponds to expression levels in untreated cells. Asterisks mark statistical significance of fold changes based on ANOVA. **B** ChIP-qPCR analysis of downregulated target genes following immunoprecipitation of p53 in treated and untreated HepG2 and Ls174 cells. *Y-*axis shows fold change compared to the chromatin of control loci; axis range matches the one of Fig. 4B for direct comparison. **C** Protein network interaction of the top 20 up-regulated and top 20 down-regulated genes in HepG2 cells **D** Protein network interaction of the top 20 up-regulated and top 20 down-regulated genes in Ls174 cells. Nodes correspond to proteins, edges indicate known (cyan:database curated, purple:experimentally determined) or predicted (green:gene neighborhood, red:gene fusions, blue:protein co-occurence, black:gene co-expression) protein–protein interactions

**Fig. 6:**
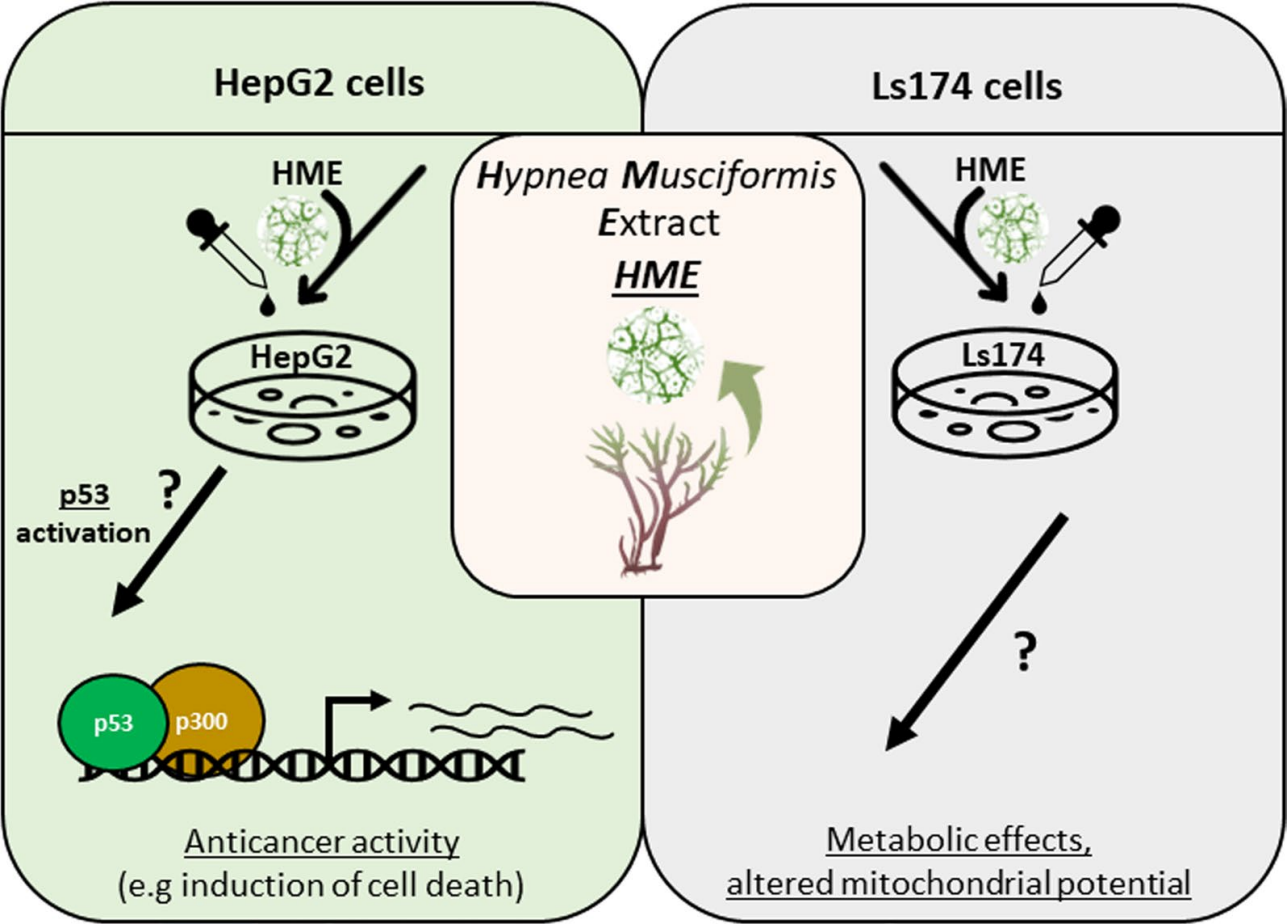
Graphical summary of the observed effects in HepG2 and Ls174 cancer cells following treatment with HME. HME triggers a p53-mediated anti-cancer response in HepG2 liver cancer cells, while it is more associated with metabolic effects in Ls174 colon cancer cells at the molecular level. The exact mechanism of p53 activation or the molecular cause of the metabolic effects are unknown

Chromatin interaction of p53 often results into recruitment of co-activators, such as p300, which subsequently primes target genes for transcription. Indeed, ChIP-western analysis confirmed the interaction between p53 and p300 on chromatin (Fig. 4C), suggesting that p300 could also be involved in the observed p53-mediated transcriptional response of HepG2 cells. We therefore expanded our ChIP-qPCR approach to test whether p300 is also recruited at the chromatin of the validated target genes. Indeed, p300 not only was significantly enriched at the chromatin of the upregulated target genes compared to control chromatin (Fig. 4D; Additional file 1: Table S7) but was also significantly enriched at the chromatin of treated compared to untreated HepG2 cells (Additional file 1: Table S8). Of note, we have also confirmed that several other genes were significantly downregulated not only in HepG2 but also in Ls174 cells (Fig. 5A; Additional file 1: Table S5). Consistent with the activating role of p53, we did not detect significant enrichment of this transcription factor at the chromatin of down-regulated targets either in HepG2 or in Ls174 cells (Fig. 5B). Finally, network analysis of the top DE genes (Additional file 1: Table S9) revealed statistically significant protein interactions in both cell lines that could be indicative of a coordinated functional response that follows HME treatment (Fig. 5C, D; Additional file 1: Table S10). In conclusion, our RT- and ChIP-qPCR analysis confirmed the observed p53-mediated response in HME-treated HepG2 cells and linked it with increased recruitment of p53 and p300 at the chromatin of the upregulated loci, as part of an overall anti-cancer response that was observed in this cell line, in contrast to the Ls174 cells in which HME treatment was mostly associated with metabolic effects (Fig. 6).

## Discussion

Previous studies have shown that seaweed extracts exhibit anti-cancerous properties in human cell lines [35]. The extracts from various seaweed species have been shown to inhibit key processes in cancer development and metastasis, such as cell proliferation [36–38], anti-inflammation [39–41], migration [42, 43], as well as induction of apoptosis [44–46]. Recently, we have reported that treatment with the extracts of several seaweed species exhibits anti-cancerous and anti-proliferation properties in HepG2 cells [47]. These observations agree with our current work findings, suggesting that extracts from *H. musciformis* can serve as a source of anticancer agents, especially in liver cancer cells.

Specifically, in HepG2 liver cancer cells our transcriptome and proteome analysis revealed transcriptional changes that alter biological functions associated with the promotion of apoptosis and cell cycle arrest. Induction of apoptosis in both cancer cell lines was also confirmed by Annexin V detection using flow cytometry analysis. As known, apoptosis is one of the major chemopreventive mechanisms of natural products [48]. Although HME-induced significant apoptosis in both cancer cell lines, apoptotic cells were not the majority of total cells at any time point of treatment, suggesting that HME may induce cancer cell death through apoptosis along with other programmed cell death forms (e.g., ferroptosis, necroptosis and pyroptosis) or autophagy. Interestingly, Ghose et al., have recently shown that *H. musciformis* extract inhibited MCF-7 breast cancer cell growth through autophagy besides apoptosis [12].

The two main apoptotic pathways are the mitochondrial (aka intrinsic) pathway and the death receptor (aka extrinsic) pathway [49]. We evaluated the effects of HME treatment on mitochondrial damage in cancer cells by measuring MMP. The results showed that HME-induced hyperpolarization in liver cancer cells, while in intestinal cancer cells hyperpolarization was also followed by depolarization. Mitochondrial hyperpolarization and depolarization indicate destabilization of mitochondrial metabolism and are considered signs of pro-apoptosis and ultimate apoptosis, respectively, in cancer cells [50].

Moreover, we identified alterations in inflammatory responses linked with neutrophil degranulation, IL-1 signaling and suppression of NF-kB signaling. Inflammation is considered to be among the driving forces of carcinogenesis [51]. Our results regarding inflammation are in agreement with recent studies revealing the anti-tumoral effect of seaweed-derived extracts in HepG2 liver cancer cells [52]. In addition, Vieira de Brito et al. [53] have reported that sulfated polysaccharides from *H. musciformis* inhibited inflammation in mice through mitigation of neutrophil migration and IL-1β levels mediated by the nitric oxide (NO) pathway.

At the molecular level, we have identified hundreds of DE genes after treatment of both cell lines with HME. We were unable to detect an overall significant correlation between our transcriptome and proteome DE genes/proteins, respectively. Low level of agreement between gene expression and protein abundance is not unusual and has been observed in previous studies with cancer cell lines [54, 55]. Such deviations could be partly explained by the accumulation of mutations in coding regions throughout the cancer genome [56], as well as to the occurrence of post-translational modifications that affect protein stability and downstream regulatory activity. However, technical experimental factors, such as the sensitivity of the two detection methods (3′ mRNA sequencing vs LS MS/MS), sample pre-processing (e.g., library amplification vs protein trypsinization etc.) together with post-detection methods of data normalization are most likely to contribute to the observed discrepancy. Furthermore, the HM extract itself may be capable of inducing transcriptional responses that subsequently get amplified (e.g., when the expression of transcription factors is deregulated) or quenched (when kinases and phosphatases are deregulated) at the translational level. Alternatively, distinct extract compounds could act separately at the transcriptional and translational level. Nevertheless, it is worth noting that at the functional level the proteo-transcriptomic gene ontology results fully agree with each other (Figs. 2F, 3F, as well as Fig. 2C, F) in both cell lines, suggesting the existence of a common functional footprint of the HME that initiates at the transcriptional and diffuses into the translational level. For example, our gene ontology results revealed that treatment of Ls174 colon cancer cells with HME triggers a transcriptional and protein response that deviates from the observed molecular effects in liver cancer cells. Moreover, the results of the phenotypic assays supported the anticancer activity of HME through cancer cell type-specific mechanisms. More specifically, in Ls174 cells, HME-induced apoptosis through mitochondrial pathway but, unlike HepG2 cells, that response was p53 independent, even though both cell lines carry wild-type p53 alleles [57, 58]. This cell type-specific activity of anticancer compounds is not unusual since altered responses to seaweed extracts between different human cancer lines have been reported in the past. For example, isolated extracts from *Coccophora langsdorfii* exhibited anti-cancerous activity in melanoma cell lines but showed weak responses in breast cancer cells [59]. In addition, seaweed extracts showed cytotoxic effects in breast cancer cells, but reduced cytotoxicity in HCT-116 colon cancer cells [60]. Of note, even the response of extracts from related seaweed species was different in breast cancer cells [61]. Since our experimental setup included treatment with a single dose and harvesting at a single timepoint, we cannot exclude the possibility that the observed differences between the two transcriptomes and proteomes are due to differential kinetics and/or dosage requirements in the response of these cells to the extract. Nevertheless, we were able to detect a statistically significant number of mutually affected genes that responded to HME treatment in a unidirectional manner between the two cell lines. Future analysis can focus on prolonged treatments of these cells with various doses of cell extracts to confirm or exclude convergence of molecular responses in these cellular backgrounds.

Our GO analysis suggests that the transcriptional and proteome alterations in Ls174 cells were predominantly associated with various metabolic changes (e.g., PPAR signaling, AMPK signaling) including but not limited to phospholipid and cholesterol metabolism as well as glucoprotein (e.g., N-Glycan and mucin type O-glycan biosynthesis) and isoprenoid (e.g., C5 isoprenoid) biosynthesis. As known, cancer metabolism exhibits significant differences from that of normal cells [62]. For example, cancer cells present increased aerobic glycolysis, which is a hallmark of the Warburg effect, thus disturbance of cancer metabolism may result in growth inhibition [62]. Moreover, our observations agree with previous studies highlighting the anti-diabetic and anti-obesity activity of various seaweed extracts. Various compounds from the red seaweed *Gracilaria opuntia* suppressed hyperglycemic response via the deactivation of α-glucosidase, a-amylase, and dipeptidyl peptidase-4 [63]. Other seaweed compounds regulated glucose uptake [64] or exhibit anti-diabetic properties [65]. Seaweed metabolic effects are also associated with lipid metabolism. For example, the red seaweed *Grateloupia elliptica* extracts reduced adipogenic proteins expression, total cholesterol and serum triglycerides contents in mice [66]. The red seaweed *Plocamium telfairiae* extracts also demonstrated anti-obesity ability via reduction of fat accumulation and suppression of major adipogenesis factors [67]. Other studies confirmed the anti-obesity effects of seaweed extracts in adipocytes [68]. Taken together, these reports agree with our observations in Ls174 cells, while our proteomic and transcriptomic data provide specific genes that mediate some of these responses at the molecular level.

In terms of molecular function, seaweed extracts have been shown to promote their anti-cancer activities through activation of several pathways and regulators. For example, polysaccharide extracts from the brown seaweed *Sargassum fusiforme* activate secretion of IL-1 and TNF-α, which in turn trigger apoptosis in HepG2 bearing mice [69]. Deactivation of PI3K/AKT signaling, another known determinant of apoptosis, was mediated by the seaweed-derived fucoidan polysaccharide triggering apoptosis in prostate cancer cells [70]. Fucoidan have been shown to independently trigger apoptosis through the ROS-mediated mitochondrial pathway in hepatoma cells [71]. Cell cycle inhibition and more specifically G0/G1 arrest has also been associated with fucoidan treatment of B large lymphoma cells [71]. p53 has been recently shown to mediate anti-cancer effects of *Sargassum* extracts in several cancer cell lines, including HepG2 [72].

Following these observations, our analysis in HepG2 cells highlighted a p53-mediated response upon treatment with the seaweed extract and linked it to the upregulation of known p53 targets in that cellular background. Our results are in agreement with previous studies demonstrating that ceramides (i.e., a family of lipids) from *H. musciformis* decreased Ehrlich ascites carcinoma in mice through increased expression of p53 and other pro-apoptotic molecules (e.g., Bax and caspase 3, [9]). Among the p53 targets that exhibit increased expression according to our findings, NFKBIA has been shown to suppress the activity of NF-κB signaling [73]. Since NF-κB antagonizes p53 [74], its inhibition by the upregulated NFKBIA would further enhance our observed p53 signature in HepG2 cells. Interestingly, NF-κB was not responsible for *H. musciformis* extract-induced apoptosis in MCF-7 breast cancer cells [12]. Nevertheless, other seaweed extracts have also been reported to regulate cancer progression through attenuation of the p53-NF-kB antagonistic axis [75].

BBC3/PUMA was another upregulated target of p53 in our HepG2 results. This protein is a central mediator of p53 apoptotic responses, through binding to anti-apoptotic Bcl-2 family members and induction of mitochondrial dysfunction and caspase activation [76]. Activation of the cellular stress protein GADD45a, which was also among our upregulated targets, has been associated with p53 mediated DNA repair [77]. p53 also regulates CDKN1A, which in turn binds to and inhibits the activity of cyclin-dependent kinase 2 or cyclin-dependent kinase 4 complexes, and thus functions as a regulator of cell cycle progression at G1 [78]. Interestingly, we have observed upregulation of VEGFA expression, that is linked with neo-angiogenesis. This disagrees with previous studies showing that ceramides from *H. musciformis* decreased VEGFB serum levels in Ehrlich ascites carcinoma mouse model [9]. Although originally it was shown that VEGFA expression is indirectly downregulated by p53 [79, 80], the interplay between p53 mediated regulation of VEGFA is more complex and can also result into upregulation of the latter [81]. However, since the levels of p53 binding in the chromatin of VEGFA are low, we certainly cannot exclude p53-independent modes of direct or indirect regulation as a possible explanation for our observed VEGFA upregulation.

The elucidation of the specific molecular mechanisms through which anticancer compounds exert their activity is a powerful tool for their utilization as targeted compounds for personalized prevention [18, 19]. Thus, the importance of identifying p53 signaling among the inhibitory mechanisms of HME function against liver cancer cell growth suggests that HME may be more appropriate as chemopreventive agent in subjects without carrying mutations in the p53 gene. In addition, our identification of metabolic pathways disturbed by HME in intestinal cancer cells may lead to its use for targeting specific molecules.

The present study investigated for the first time the induced changes of *H. musciformis* extract treatment in whole transcriptome and proteome expression of cancer cells. Specifically, in this work we have characterized the transcriptome and proteome of liver and colon cancer cells after treatment with HME, providing several examples of deregulated p53 target genes, which can explain part of our observed phenotypes. Moreover, we linked p53 presence at the chromatin of upregulated target genes with the recruitment of the p300 co-activator, although we cannot exclude the inverse scenario in which p300 recruits p53 on the target chromatin. p53 has been reported to facilitate target chromatin accessibility through enhancement of p300/CBP autoacetylation, leading to histone acetylation [82, 83]. Future experiments should further dissect this observed p53/p300 interplay, coupled to the analysis of the *H. musciformis* extracts with the aim of isolating and characterizing the responsible bioactive compounds that mediate the observed p53 response. Since our proteomic approach did not detect alterations in p53 overall levels, future efforts should also focus on explaining the molecular signaling that underpins these responses.

Of particular interest would be the characterization of p53 phosphorylation or the phosphorylation of its downstream effectors, through phosphoproteomic analysis after treatment of cancer cells and/or murine models with HME. In addition, it would be of equal interest to in-depth characterize the metabolic effects that were observed in Ls174 colon cancer cells, with metabolomic approaches. Our results along with the aforementioned future studies will substantially help to elucidate the molecular mechanisms accounting for HME’s chemopreventive activity, which is a prerequisite for using them as food supplements or as biofunctional foods.

## Conclusion

In conclusion, this functional genomics study is the first to provide functional insights regarding the anti-cancer properties of *H. musciformis* marine seaweed extracts in liver and colon cancer cells. The results link the observed tumor-suppressive phenotypes in response to extract treatment with extensive transcriptome and proteome responses. Furthermore, the study provides specific evidence regarding the molecular mechanisms that govern the induction of cell death in liver cancer cells, linking them with chromatin interactions and p53-mediated transcriptional responses. These results highlight both the nutritional value of marine seaweeds and the necessity of thoroughly evaluating their benefits at the molecular level on the road of developing personalized patterns of nutrition.

## Supplementary Information

**Additional file 1. **This excel sheet contains additional statistical information organised and described as supplementary tables 1 to 10 in main text.

**Additional file 2. Figs. 1**: Flow cytometry analysis. **A**) Annexin V-PE/7-AAD FACS dot plots following HME treatment for 0.5, 1, 2, 4, 8, 16 and 24 h in Ls174 cells. **B**) Annexin V-PE/7-AAD FACS dot plots following HME treatment for 0, 0.5, 1, 2, 4, 8, 16 and 24 h in HepG2 cells. **C**) Histograms for FACS assessment of mitochondrial membrane potential (MMP) in Ls174 cells after treatment with HME for 0.5, 1, 2, 4, 8, 16 and 24 h (X-axis: FL2 height; Y-axis: Cell counts) **D**) Histograms for FACS assessment of mitochondrial membrane potential (MMP) in HepG2 cells after treatment with HME for 0.5, 1, 2, 4, 8, 16 and 24 h (X-axis: FL2 height; Y-axis: Cell counts).

**Additional file 3. Figs. 2**: Transcriptome and proteome analysis in Ls174 cells. **A**) Heatmap corresponding to transcriptional change in Ls174 cells with and without treatment. Gene expression was log2 transformed and shown as normalized z-score. **B**) Volcano plot illustrating statistically significant differentially expressed genes in Ls174 cells following treatment with the seaweed extract. Y-axis represents p-value after − log10 transformation, x-axis corresponds to log2 transformed fold change. **C**) Dot plot summarizing gene ontology results of biological function for the statistically significant DE genes in Ls174. Dot size corresponds to number of DE genes and colour corresponds to statistical significance for each cellular function. **D**) Heatmap corresponding to protein change in Ls174 cells with and without treatment. Protein levels log2 transformed and shown as normalized z-score. **E**) Volcano plot illustrating statistically significant alterations of protein levels in Ls174 cells following treatment with the seaweed extract. Y-axis represents p-value after − log10 transformation, x-axis corresponds to log2 transformed fold change. **F**) Dot plot summarizing gene ontology results of biological function for the statistically significant alterations in protein levels in Ls174. Dot size corresponds to number of DE proteins and colour corresponds to statistical significance for each cellular function.

## Data Availability

The RNA-seq data that support the findings of this study are openly available in GEO functional genomics data repository with the dataset identifier GSE228578. The mass spectrometry proteomics data have been deposited to the ProteomeXchange Consortium via the PRIDE [84] partner repository with the dataset identifier PXD041686. The authors confirm that the data supporting the findings of this study are available within the article [and/or] its supplementary materials.

## Abbreviations

HME: *Hypnea musciformis* Extract
MMP: Mitochondrial membrane potential
Annexin V-PE: Annexin V-Phycoerythrin
7-AAD: 7-Amino-actinomycin D
TNA: Total nucleic acid
WB: Western blot
ChIP: Chromatin immunoprecipitation
MDS: Multi-dimensional scaling
DE: Differentially expressed
GO: Gene ontology
