# Supplementary figures and images for: Transcriptome and proteome analysis reveals the anti-cancer properties of Hypnea musciformis marine macroalga extract in liver and intestinal cancer cells
Source: Hum Genomics. 2023 Jul 31;17:71. doi: 10.1186/s40246-023-00517-0 (PMC10388463; doi:10.1186/s40246-023-00517-0)

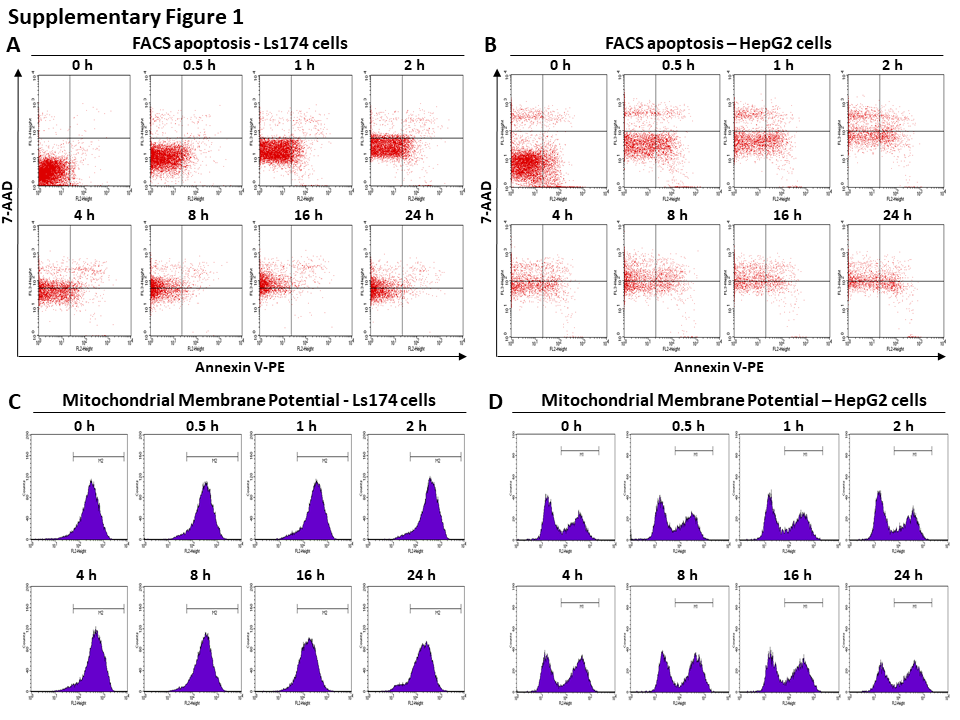

Supplement: Supplementary file 2 — Additional file 2. Figs. 1: Flow cytometry analysis. A) Annexin V-PE/7-AAD FACS dot plots following HME treatment for 0.5, 1, 2, 4, 8, 16 and 24 h in Ls174 cells. B) Annexin V-PE/7-AAD FACS dot plots following HME treatment for 0, 0.5, 1, 2, 4, 8, 16 and 24 h in HepG2 cells. C) Histograms for FACS assessment of mitochondrial membrane potential (MMP) in Ls174 cells after treatment with HME for 0.5, 1, 2, 4, 8, 16 and 24 h (X-axis: FL2 height; Y-axis: Cell counts) D) Histograms for FACS assessment of mitochondrial membrane potential (MMP) in HepG2 cells after treatment with HME for 0.5, 1, 2, 4, 8, 16 and 24 h (X-axis: FL2 height; Y-axis: Cell counts). [file 40246_2023_517_MOESM2_ESM.tif]

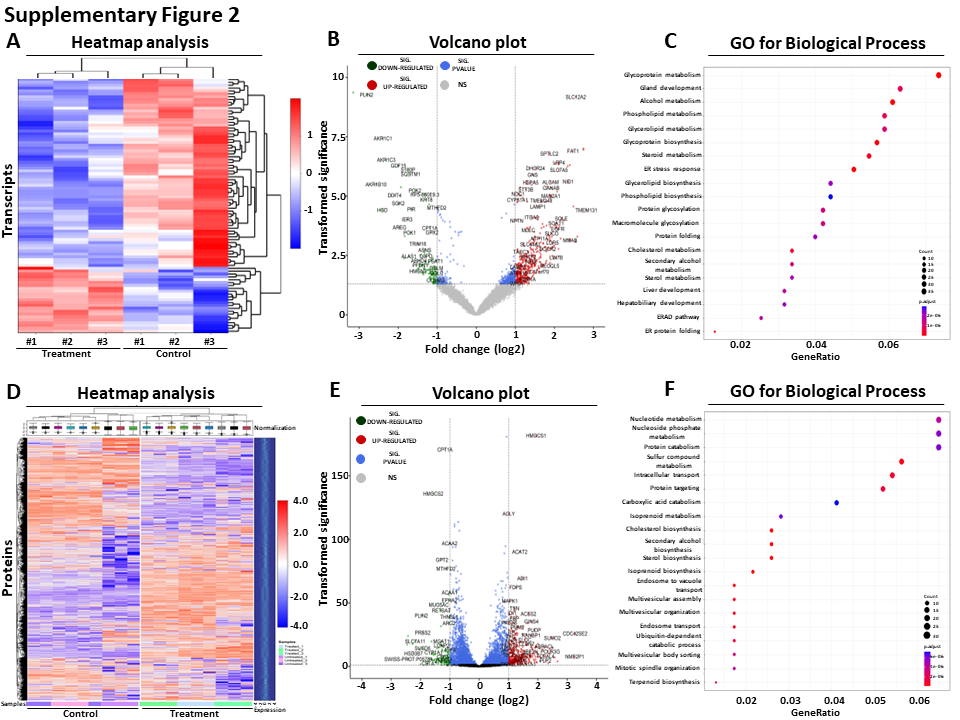

Supplement: Supplementary file 3 — Additional file 3. Figs. 2: Transcriptome and proteome analysis in Ls174 cells. A) Heatmap corresponding to transcriptional change in Ls174 cells with and without treatment. Gene expression was log2 transformed and shown as normalized z-score. B) Volcano plot illustrating statistically significant differentially expressed genes in Ls174 cells following treatment with the seaweed extract. Y-axis represents p-value after − log10 transformation, x-axis corresponds to log2 transformed fold change. C) Dot plot summarizing gene ontology results of biological function for the statistically significant DE genes in Ls174. Dot size corresponds to number of DE genes and colour corresponds to statistical significance for each cellular function. D) Heatmap corresponding to protein change in Ls174 cells with and without treatment. Protein levels log2 transformed and shown as normalized z-score. E) Volcano plot illustrating statistically significant alterations of protein levels in Ls174 cells following treatment with the seaweed extract. Y-axis represents p-value after − log10 transformation, x-axis corresponds to log2 transformed fold change. F) Dot plot summarizing gene ontology results of biological function for the statistically significant alterations in protein levels in Ls174. Dot size corresponds to number of DE proteins and colour corresponds to statistical significance for each cellular function. [file 40246_2023_517_MOESM3_ESM.tif]
